# Evolution of the Auxin Response Factors from charophyte ancestors

**DOI:** 10.1371/journal.pgen.1008400

**Published:** 2019-09-25

**Authors:** Raquel Martin-Arevalillo, Emmanuel Thévenon, Fanny Jégu, Thomas Vinos-Poyo, Teva Vernoux, François Parcy, Renaud Dumas

**Affiliations:** 1 Laboratoire de Reproduction et Développement des Plantes, Univ. Lyon, ENS de Lyon, UCB Lyon1, CNRS, INRA, Lyon, France; 2 Univ. Grenoble Alpes, CNRS, CEA, INRA, IRIG-DBSCI-LPCV, Grenoble, France; University of North Carolina, UNITED STATES

## Abstract

Auxin is a major developmental regulator in plants and the acquisition of a transcriptional response to auxin likely contributed to developmental innovations at the time of water-to-land transition. Auxin Response Factors (ARFs) Transcription Factors (TFs) that mediate auxin-dependent transcriptional changes are divided into A, B and C evolutive classes in land plants. The origin and nature of the first ARF proteins in algae is still debated. Here, we identify the most ‘ancient’ ARF homologue to date in the early divergent charophyte algae *Chlorokybus atmophyticus*, CaARF. Structural modelling combined with biochemical studies showed that CaARF already shares many features with modern ARFs: it is capable of oligomerization, interacts with the TOPLESS co-repressor and specifically binds Auxin Response Elements as dimer. In addition, CaARF possesses a DNA-binding specificity that differs from class A and B ARFs and that was maintained in class C ARF along plants evolution. Phylogenetic evidence together with CaARF biochemical properties indicate that the different classes of ARFs likely arose from an ancestral proto-ARF protein with class C-like features. The foundation of auxin signalling would have thus happened from a pre-existing hormone-independent transcriptional regulation together with the emergence of a functional hormone perception complex.

## Author summary

Plants transition from water to land was determining for the history of our planet, since it led to atmospheric and soil condition changes that promoted the appearance of other life forms. This transition initiated around 1 billion years ago from a Charophyte algae lineage that acquired features allowing it to adapt to the very different terrestrial conditions. Land plants coordinate their development with external stimuli through signalling mechanisms triggered by plant hormones. Therefore, evolution of these molecules and their signalling pathways likely played an important role in the aquatic to terrestrial move. In this manuscript we study the origin of auxin signalling, a plant hormone implicated in all plant developmental steps. Our studies suggest that out of the three families of proteins originally proposed to trigger auxin signalling in land plants, only one existed in Charophyte ancestors as a likely transcriptional repressor independent of auxin. We show that despite millions of years of evolution, this family of proteins has conserved its biochemical and structural properties that are found today in land plants. The results presented here provide an insight on how hormone signalling pathways could have evolved by co-opting a pre-existing hormone-independent transcriptional regulatory mechanism.

## Introduction

**Fig 1:**
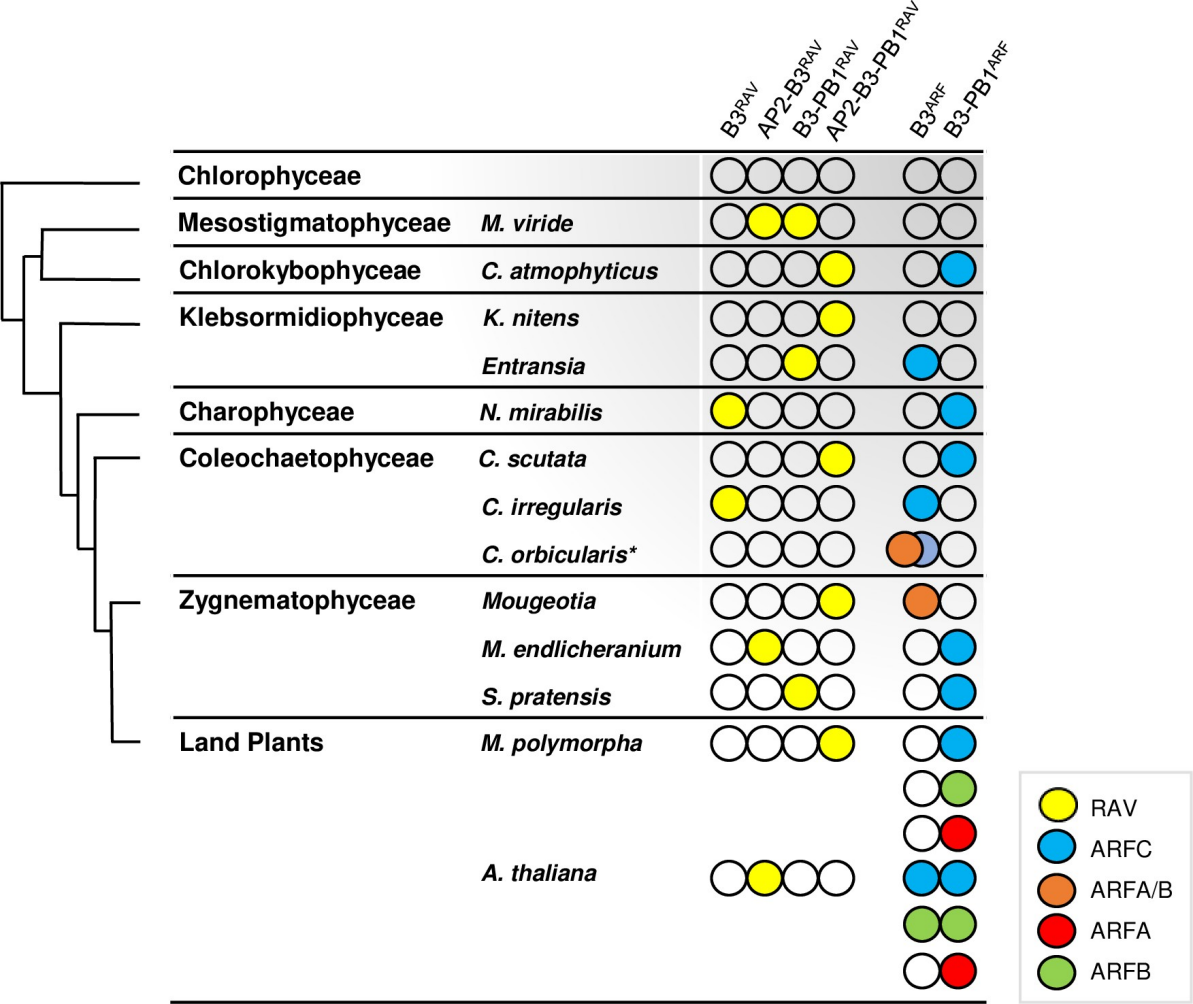
Charophytes B3- and PB1-domain containing proteins along evolution of charophytes algae and land plants. On the left, an illustrated view of charophytes evolutionary line from chlorophytes to land plants adapted from [9,42]. Although some publications placed Mesostigmaphoticae and Chlorokybophycae in a unified clade large differences in sequences and morphological traits argues for two different clades [2,6,42,43]. The presence of class C, A/B, A or B ARFs is indicated by blue, orange, red or green circles whereas yellow circles correspond to RAV proteins. Identities of proteins reported in the figure are shown in Supplementary S2 Table.

Charophytes diverged from chlorophyte algae more than a billion years ago (y.a.) and led to land plants emergence around 450 million y.a. [1–5]. “Early divergent” clades display a range of body complexity going from unicellular algae in Mesostigmatophyceae and Chlorokybophyceae, to multicellular filaments in Klebsormidiophyceae (Fig 1; S1 Fig) [6,7]. “Late divergent” clades include Charophyceae and Coleochaetophyceae that share features with land plants (S1 Fig), [8,9] but also Zygnematophyceae, that despite their simple structure are considered sisters to land plants according to recent phylogenetic studies [10,11].

Given the importance of the phytohormone auxin in plant development, the acquisition of its signalling pathway allowing for auxin-dependent changes in transcription is thought to have been a milestone in the water-to-land transition [1]. In land plants, this signalling pathway, called the Nuclear Auxin Pathway (NAP), relies on three main protein families: TIR1/AFB (Transport Inhibitor Response 1/Auxin Signalling F-box) co-receptors, Aux/IAA transcriptional repressors (Auxin/Indole-3-Acetic Acid Protein) and ARF (Auxin Response Factors) Transcription Factors (TFs) [12,13]. ARFs have been classified into three evolutive classes, A, B and C. Class A includes activator ARFs whereas classes B and C contain repressor ARFs [14]. ARF interaction with DNA is mediated by their B3 domain (B3^ARF^). Such domain is also present in ABI3 (Abscisic Acid insensitive 3, B3^ABI3^) and RAV (Related to ABI/VP1, B3^RAV^) plant TFs but with different DNA binding specificities [15,16]. In the ARF family, the B3 domain is embedded in a larger N-terminal DNA Binding Domain (DBD) that includes a Dimerization Domain (DD). As dimers, ARFs bind double AuxREs (Auxin Response Elements) sites arranged in three possible orientations: Direct Repeat (DR), Everted Repeat (ER) and Inverted Repeat (IR) (S2 Fig) [1,17–19].

In charophyte algae and the bryophyte *Marchantia polymorpha*, the B3^RAV^ and B3^ARF^ domains are often associated with a C-terminal PB1 oligomerization domain, a landmark of most ARF TFs in higher plants but that was lost by RAV TFs from tracheophytes [1,20]. This shared B3 + PB1 domain composition led to the initial hypothesis that ARF could have arisen from RAV [21]. In the NAP, the PB1 domain mediates ARF homo-oligomerization and hetero-oligomerization with Aux/IAAs [22]. Under low auxin concentrations, Aux/IAAs bind activator ARFs through their PB1 domain [23–26] and recruit TOPLESS (TPL)/TOPLESS-RELATED co-repressors, leading to the formation of a repressor complex on regulatory sequences of auxin-responsive genes [27]. When auxin levels increase, the hormone-mediated interaction between Aux/IAA and TIR1/AFB leads to Aux/IAA proteasomal degradation, unlocking activator ARFs and inducing transcription [28,29]. Most class B and C ARF members have limited interaction capacities with Aux/IAAs [30–32] and are proposed to regulate auxin transcriptional responses in an auxin-independent manner, possibly by competitive binding with class A ARFs on DNA regulatory sequences [33,34]. Since some of class B and C ARFs can interact directly with TPL, formation of co-repressor complexes was proposed as another possible mechanism for transcriptional repression of auxin target genes [34–36].

The NAP was established at the beginning of land plants history. In the bryophyte *M*. *polymorpha* for example, the 3 families of NAP proteins are present (with one member of each ARF class) as well as the TPL co-repressor [37,38]. Recent studies showed the existence of two ARF subfamilies in charophytes, class C and class A/B [20], but the absence of functional TIR1/AFB and Aux/IAAs suggested that a fully functional NAP did not exist before land plants [1,20,37,39–41]. How these ancestral components evolved to form the land plants NAP remains an open question. Through the structural, biochemical and phylogenetic characterisation of a proto-ARF from an early divergent charophyte we set a scenario of how the co-option of ancestral mechanisms of transcriptional control possibly led to the evolution of hormone signalling pathways in plants.

## Results

### Identification of proto-ARF and proto-RAV in early divergent charophytes

To understand the evolution of ARFs, we first characterized the biochemical properties of proto-ARFs and closely related proto-RAVs from early divergent charophytes. We searched for B3 homologues in charophyte transcripts databases (OneKp and Marchantia.info) [11,44] and classified them as B3^RAV^ or B3^ARF^, depending on the residues signature of their predicted DBDs (S1 Table) [45]. B3^RAV^ domains were frequently associated with an APETALA2 (AP2) domain and/or PB1 domains in the basal charophyte *Mesostigma viride* and all later clades (Fig 1; S2 Table). *M*. *viride* also has an ARF homologue (GBSK01006108.1) devoid of a PB1 domain [1]. Its DBD was reliably modelled as an ARF (100% confidence with AtARF1 [46,47]), but it lacks most residues involved in the interaction with AuxREs (S3 Fig) and thus does not qualify as a functional ARF. The proto-ARF of the earliest diverging green Charophyte algae with predicted functional B3^ARF^ and PB1 domains was found in *C*. *atmophyticus*. Other ARF homologues were also present in all later diverging clades (Fig 1; S3 Fig).

### DNA binding specificities and oligomerization potential of proto-RAV and proto-ARF

**Fig 2:**
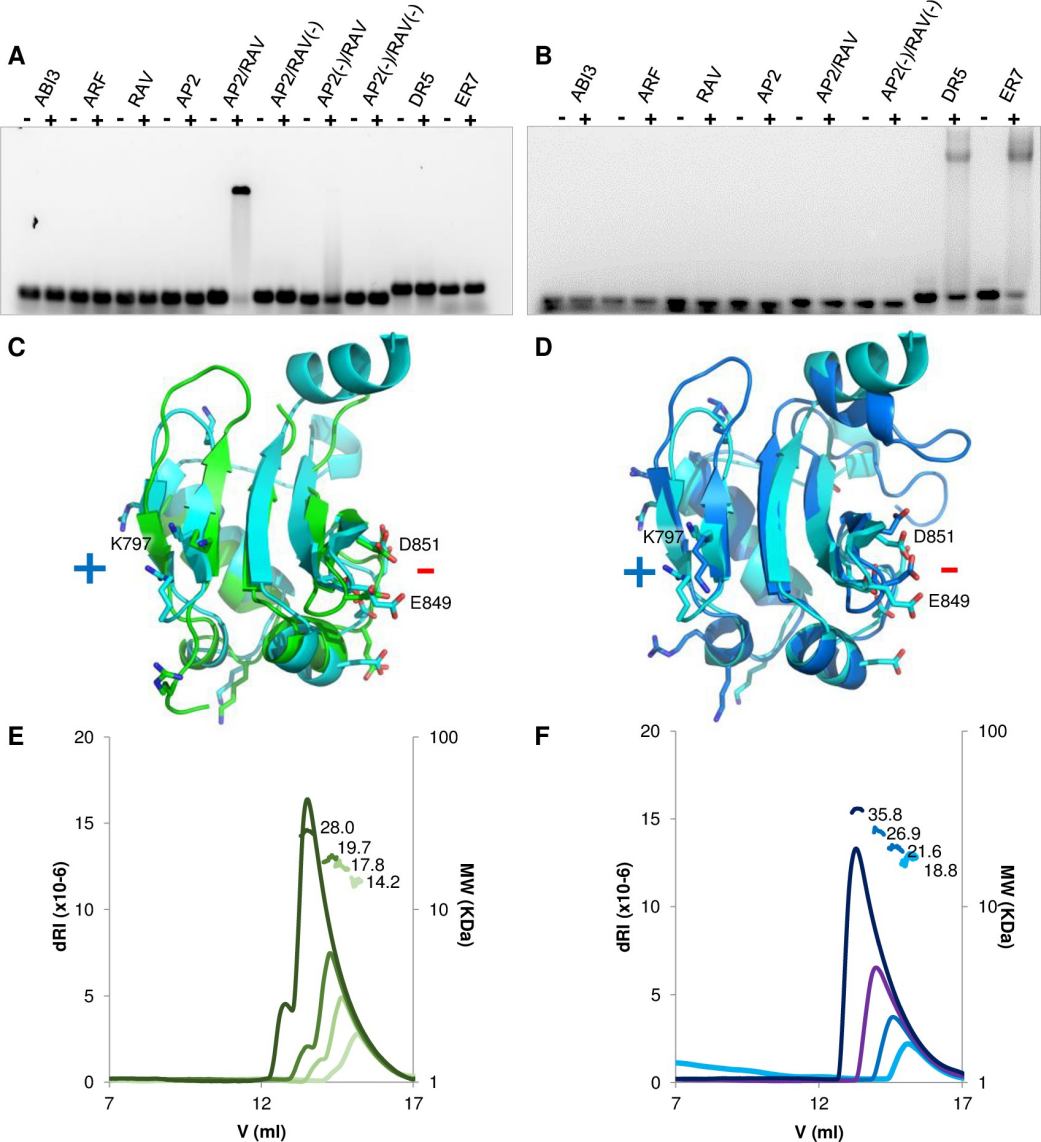
Proto-RAV and proto-ARF biochemical properties. (A), KnRAV-DBD (AP2/B3 domains) or (B), Ca-ARF-DBD interactions in EMSA with specific single binding sites for different B3 families (ABI3, ARF and RAV) [45], AP2, a composite site for AP2-B3/RAV family, AP2(-)/RAV mutated on AP2, AP2/RAV(-) mutated on RAV, AP2(-)/RAV(-) mutated on both AP2 and RAV, DR5 (Direct Repeats AuxRE spaced by 5 nucleotides) and ER7 (Everted Repeats spaced by 7 nucleotides) ARF motifs. DNA sequences are indicated in S8 Table. Proteins added at 0 and 0.5 μM. (C-D), Structure models for KnRAV-PB1 (C, green) and CaARF-PB1 (D, blue) superposed to AtARF5-PB1 structure (cyan) (PDB code 4CHK [23]). Conserved residues indicated refer to AtARF5-PB1 structure. Positive and negative signs indicate potential interaction surfaces for oligomerization. (E), SEC-MALLS KnRAV-PB1 molecular weight determination for four protein concentrations (from dark to light green: 5 mg/mL, 2.5 mg/mL, 1.25 mg/mL and 0.625 mg/mL). (F), SEC-MALLs CaARF-PB1 molecular weight determination (from dark to light blue: 5 mg/mL, 2.5 mg/mL, 1.25 mg/mL and 0.625 mg/mL.

We determined the properties of “ancestral” RAV and ARF proteins, focusing on *Klebsormidium nitens* proto-RAV (containing predicted AP2, B3^RAV^ and PB1) (KnRAV, kfl00094_0070) and *C*. *atmophyticus* proto-ARF (CaARF, AZZW-2021616). The predicted B3 domains of KnRAV and CaARF display the signature residues typical of B3^RAV^ and B3^ARF^, respectively (S1 Table; S3 and S4 Figs) suggesting that their divergent DNA binding specificities were already established in charophytes. To test this hypothesis, we characterized the binding of their DBD against the canonical DNA binding sites identified in angiosperms for ABI3, RAV and ARF TFs. KnRAV specifically bound the AP2/B3^RAV^ bipartite element described for *Arabidopsis thaliana* RAV TFs (Fig 2A) [48]. CaARF interacted strongly with double AuxRE sites (DR or ER, Fig 2B) but not with a single AuxRE site suggesting that the DBD of CaARF binds DR and ER motifs as a dimer without the help of the Middle Region (MR) and the PB1 domain. Altogether, these results confirm that RAV and ARF DNA binding preferences were established in basal charophytes and maintained along evolution.

Next, we studied the oligomerization capacity of their PB1 domain. Based on AtARF5 PB1 structure [23,47], the PB1 domains of KnRAV and CaARF were modelled as type I/II PB1 with electrostatic oligomerization potential (Fig 2C and 2D). Molecular weight determination of KnRAV-PB1 and CaARF-PB1 by Size Exclusion Chromatography combined with Multi-Angle-Light Scattering (SEC-MALLS) experimentally validated that both domains form oligomeric complexes (Fig 2E and 2F) but with a lower oligomeric potential than AtARF5-PB1 (S3 Table). Charophycean algae therefore appear to possess proto-RAV and proto-ARF proteins with oligomerization potential and diverging DNA binding specificities (Fig 2A and 2B; S3 and S4 Figs).

### Evolution of ARF DNA binding specificity from early divergent charophytes to land plants

**Fig 3:**
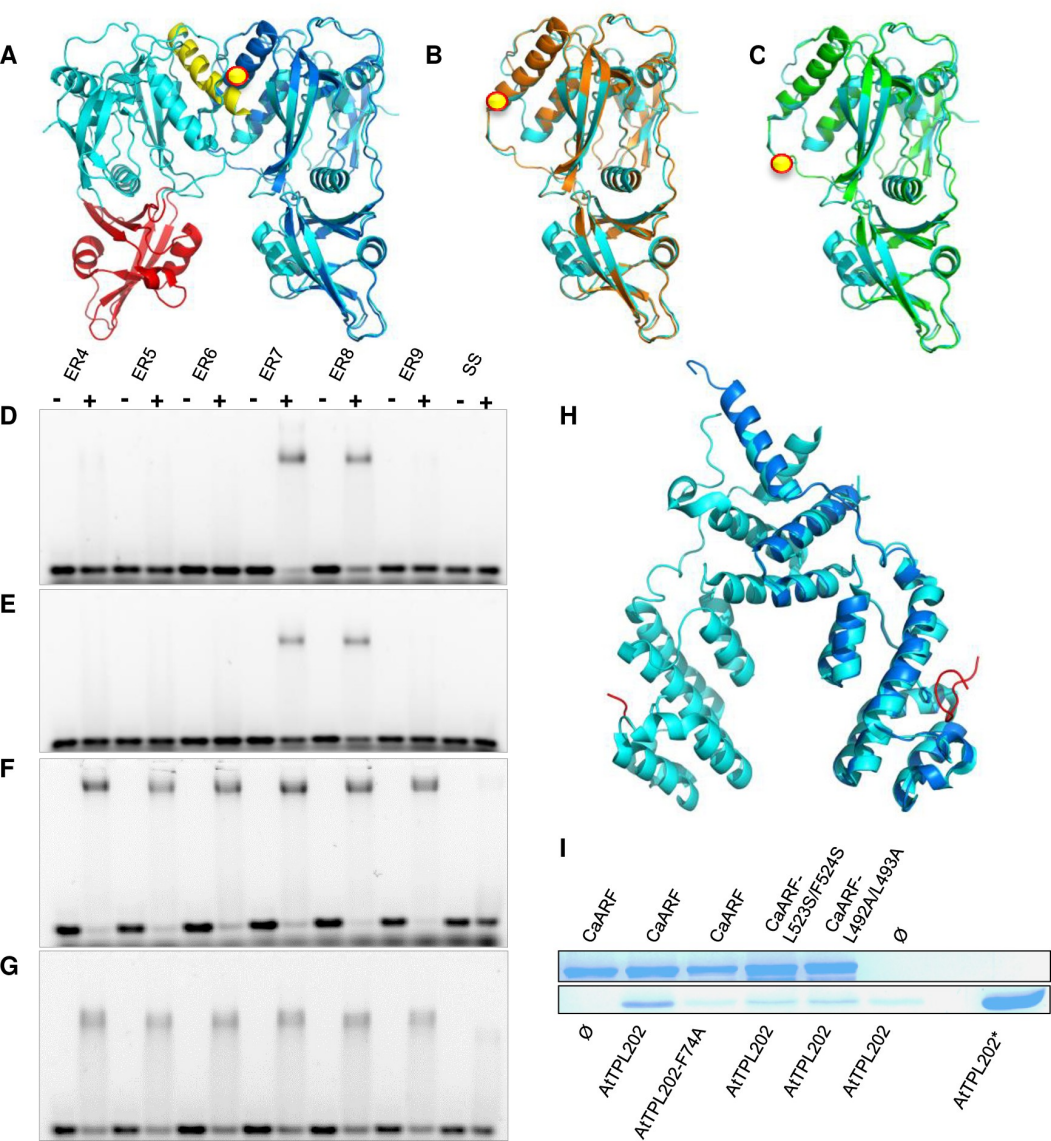
Ancestral class C ARFs exhibit different DNA specificities and TPL recruitment potential. (A), CaARF-DBD modelled structure (dark blue) superposed to ARF1 dimer (cyan with DD in yellow and B3 in red indicated on one monomer (4LDX, [46])). (B-C), Class-C MpARF3 (orange, B) and AtARF10 (green, C) DBD structure models superposed to ARF1 (cyan). Yellow dots indicate the position of the additional disordered region, not modelled. (D-G), AtARF2 (D), AtARF5 (E), CaARF (F) and AtARF10 (G) binding to ER DNA binding sites with a spacing ranging from 4 to 9 nucleotides. Positive signs indicate lanes where protein was added (AtARF2, AtARF5 and AtARF10 at 0.25 μM; CaARF at 1 μM). (H), *C*. *atmophyticus* predicted TPL (AZZW-2021890) N-ter structure model (dark blue) superposed with AtTPL N-ter structure (cyan) (5NQV, [49]). (I), Co-purification interaction assays between MBP-tagged CaARF (and mutants: CaARFmL523S/F524S; CaARFmL492A/L493A) and AtTPL202 (and mutant AtTPL202mF74A). Complexes were bound to Dextrin-Sepharose columns. SDS-PAGEs show the proteins eluted from the column. AtTPL202* is a control indicating the size of the TPL N-ter protein.

To further characterize the biophysical properties of proto-ARFs, we determined the predicted structure of CaARF DBD and showed that it was reliably modeled (99% confidence; Phyre 2) with AtARF1 and AtARF5 DBDs [46,47] except for an additional disordered region in CaARF present within the DD (Fig 3A). Similar disordered regions were found as a characteristic feature of all class C ARFs (Fig 3B and 3C; S3 and S7 Figs). In agreement with this, our phylogenetic studies position CaARF within clade C (S5 Fig). Such insertions are expected to modify class C DNA binding compared to A and B ARFs. We tested this hypothesis using ER motifs with different spacing (ER4-9). Unlike Arabidopsis AtARF2 (class B) and AtARF5 (class A) that largely prefer ER7/8 motifs (Fig 3D and 3E), CaARF showed promiscuous binding to ER4-9 but did not interact with a single AuxRE motif (Fig 3D–3F) confirming its interaction with ER motifs as a dimer. Arabidopsis class C AtARF10 behaves similarly to CaARF (Fig 3G). This shows that CaARF has a relaxed DNA specificity allowing binding to ER binding sites with various distances between the monomeric sites and that this specificity was maintained in class C ARF along plants evolution. The presence of a specific disordered region (Fig 3A–3C; S3 and S7 Figs) in class C ARF DBDs suggests a possible role in their relaxed specificity, that remains to be tested.

### Interaction with co-repressors in early divergent charophytes

As mentioned before, certain land plants ARF proteins have the capacity to interact directly or indirectly with the TPL co-repressor [35,36,50]. We wondered when in evolution this interaction was first established. Direct TPL-recruitment usually involves two different amino acid regions in the Middle Region (MR) of repressor ARFs: the EAR-motif (ERF-associated Amphiphilic Repression motif with LxLxL sequence or its LxLxPP variant) and the BRD domain (B3 Repression Domain with the K/RLFG sequence) [35,36], the BRD domain also being found in RAV proteins. CaARF-MR presents two potential repression regions with an EAR-like motif (LPLLPS, similar to LxLxPP) and a BRD domain (KLFG). Since TPL EAR-interacting-region (TPL N-terminal, TPL-N) is extremely conserved between charophytes and land plants [49,51] (Fig 3H; S8 Fig; S4 Table), we used *A*. *thaliana* TPL-N (AtTPL202) to assay the TPL/CaARF interaction. CaARF interacted with AtTPL202 in co-purification assays and this interaction was lost with AtTPL202^-F74A^, mutated in the hydrophobic EAR peptide binding groove (Fig 3I) [49]. Moreover, mutations in CaARF KLFG (CaARF^-L523S/F524S^) or LPLLPS (CaARF^-L492A/L493A^) weakened the interaction with AtTPL202, indicating that both sites might participate to TPL-N recruitment. The binding of the BRD domain of CaARF differs from that of the RAV1 of *A*. *thaliana* which interacts with the C-terminal part of TPL [52], suggesting different TPL recruitment mechanisms for these two protein families. The presence of similar TPL-recruitment sequences in proto-ARFs of different charophytes clades ARFs (S5 Table) suggests that they might also interact with TPL.

## Discussion

The present biochemical characterization of CaARF, a proto-ARF from an “early divergent” charophyte, identifies this protein as class C ARF, in agreement with our phylogenetic analyses (S5 Fig). Mutte et al. (2018) proposed the existence of two ARF classes in “late divergent” charophytes, C and A/B, deriving from a common ancestor that diverged in an ancient charophyte clade [20]. Based on phylogenetic analyses showing that class C ARF is sister to classes A and B, and on the identification of a *M*. *viride* sequence classified as a class A/B, Flores-Sandoval et al. (2018) proposed a similar scenario where the divergence between classes A and B and class C occurred prior to the diversification of extant streptophytes [40]. This plausible scenario, built before the identification of class C ARFs in “early divergent” charophytes, is based on an unusual *M*. *viride* sequence that does not exhibit the conserved ARF DNA binding residues (S3 Fig), and implies repeated loss of class A/B ARFs from Chlorokybophyceae to Coleochaetophyceae (S6 Fig). Further identification of class C ARFs in the “early divergent” charophytes (Klebsormidiophyceae [1] and Chlorokybophyceae (this work)) and the presence of both classes C and A/B in the “late divergent” *C*. *orbicularis* suggest a second and more parsimonious scenario in which class A/B ARF members come from an ancestral proto-ARF, belonging to class C or class C-like that existed before the emergence of “late divergent” charophytes (S6 Fig). This hypothesis implies only a few class C *ARF* gene losses in some Klebsormidiophyceae, Coleochaetophyceae and Zygnematophyceae species. Still, all these scenarios need to be taken with caution as they are based on transcriptomic datasets and could be challenged when genomic sequences become available.

When comparing C and A/B clades we found a disordered region within the predicted DD of ancestral and land plants clade C ARFs that is not present in clade A/B neither in land plants clades A and B. We speculate that during the duplication event leading to A/B emergence from clade C, the loss of this disordered sequence occurred. The DNA interaction experiments presented in this manuscript suggest that this event might have contributed to the acquisition of a more restricted DNA specificity of class A and B ARFs for ER motifs.

Apart from the similar behaviour observed for CaARF and AtARF10 when binding to DNA, ancestral clade C ARFs already presented PB1 oligomerization potential and interaction with the co-repressor TPL. The conservation of these properties along evolution is consistent with experiments conducted on Marchantia showing partial complementation of the loss of function Mp*ARF3* by class C AtARF10 [40]. Moreover, these biochemical facts are instructive on several aspects of the evolution of the NAP in plants. First, proto-ARFs being able to interact with AuxREs supports that the NAP could have co-opted sets of genes already regulated by ARFs in charophytes, as suggested in other studies [1,20,39,40]. In this context, the emergence of the A/B clade with a different DNA binding behaviour could have allowed to target a more specific set of genes. Second, proto-ARF interaction with TPL provides functional evidence for a role for class C ARFs as transcriptional repressors. Putative TPL interaction motifs are also present in proto-RAV and most proto-ARFs across charophytes, which includes class A/B ARFs. The capacity to recruit TPL co-repressors could thus be an ancestral property of RAV and ARF TFs.

**Fig 4:**
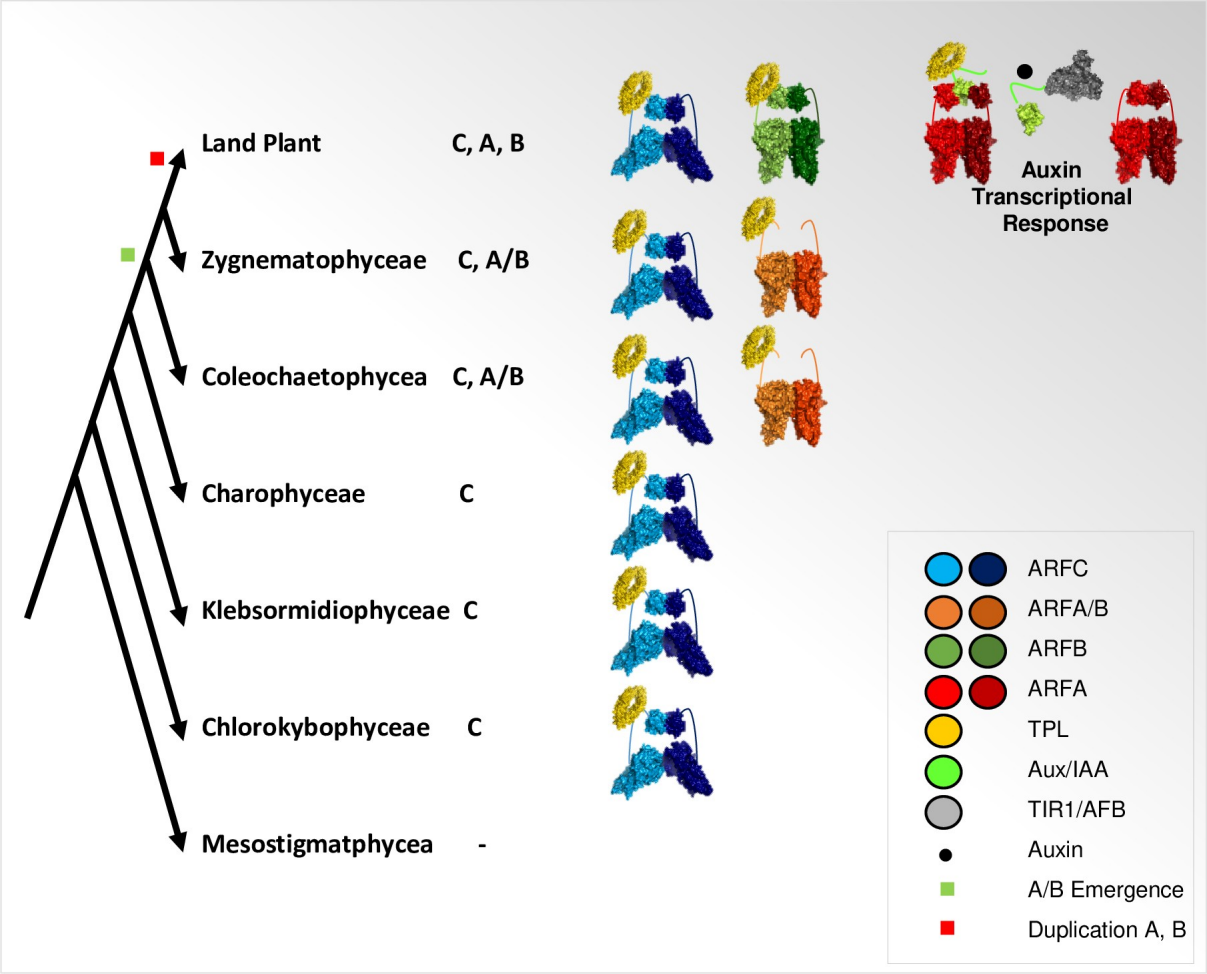
Model for ARFs and NAP evolution. First ARF homologues that appeared in plants evolutionary line are class C ARFs, found in early charophytes and in all subsequent clades till land plants. Charophycean class C ARF ancestor recognizes different AuxRE motifs as a dimer, can oligomerize via its PB1 domain and interact with the corepressor TPL. In charophyte clades that diverged later, the class A/B ARF subfamily emerged, likely from class C. This new subfamily evolved a different DNA specificity through an alternative dimerization mode. The division of A/B into classes A and B appeared only in land plants together with TIR1-AuxIAAs perception system that confers auxin sensibility to the charophycean ARF-regulatory mechanism leading to a functional NAP.

From these observations, we propose ARFs recruitment of co-repressor complexes to AuxREs promoter elements as a primitive and conserved mechanism predating the NAP. The absence of a functional TIR/AFB-Aux/IAA co-receptor [1,20,41] indicates that this primitive system was auxin-independent. These observations are consistent with a series of experiments in Marchantia showing that auxin-responsive genes show similar transcriptional responses in WT and Mp*ARF3* mutants [20,40]. Alongside the diversification of ARF DNA binding specificity, emergence of the auxin perception complex in the first land plants turned ARFs-regulated genes into auxin-responsive genes through ARF-Aux/IAA-TIR/AFB interactions evolution (Fig 4).

Our work thus allowed proposing a scenario where the evolution of the binding specificity of an ancestral TF together with the emergence of a functional hormone perception complex create a hormone signalling pathway. This scenario offers a better understanding of how hormone signalling pathways can evolve from pre-existing mechanisms of transcriptional regulation independent of any hormone signalling.

## Materials & methods

### Protein homologues search and classification

Potential homologs of the NAP components were searched by sequence homology to the corresponding NAP proteins from *M*. *polymorpha*. Blasts were done using different databases: OneKp, PlantTFDB and Marchantia.info. Due to the lack of proteomic data in charophyte organisms, we carried out tblastn. Each transcript was then translated using Expasy Translate tool. Sequences resulting from this search were classed using protein sequence alignments and phylogenetical studies. Protein sequences alignments were done with Multialin (http://multalin.toulouse.inra.fr/multalin/) and ESPrit (http://espript.ibcp.fr/ESPript/ESPript/) online tools. Phylogenetic analyses were conducted using predicted DBDs from charophyte proto-ARFs and DBDs belonging to *A*. *thaliana* and *M*. *polymorpha* ARFs. Phylogenies were done with MEGA and Phylogeny.fr software using Maximum likelihood algorithm.

### 3D structure modelling

Protein structure modelling was done with Phyre2 online tool [47]. Three-dimensional structures were visualized with PyMOL software (www.pymol.org).

### Plasmids construction for expression in *E*. *coli*

cDNA sequences coding for potential ancestors and the corresponding mutants were constructed as synthetic DNA (Thermofisher). KnRAV and CaARF (full-length, fragments (CaARF-DBD (residues 1–421), CaARF-PB1 (residues 644–750), KnRAV-DBD (residues 256–523), KnRAV-PB1 (residues 724–798)) or mutants) coding sequences were cloned into pETM40 plasmid (EMBL) that contains a MBP-tag in the N-terminal region except for PB1 domains from both proteins that were cloned into pETM11 (EMBL) that confers a N-terminal His-tag.

KnRAV and CaARF specific domains were isolated by PCR from synthetic cDNA sequences (S6 Table). Full-length ARF2, ARF5 and ARF10 were cloned into pHMGWA vectors (Addgene) containing N-terminal His-MBP-His tags.

### Protein expression and purification

All proteins were expressed in *Escherichia coli* BL21 strain. Bacteria cultures were grown with the appropriate antibiotics at 37°C until they achieved an OD_600nm_ of 0.6–0.9. Protein expression was induced with isopropyl-β-D-1-thyogalactopiranoside (IPTG) at a final concentration of 400 μM at 18°C overnight. Bacteria cultures were centrifuged, and the pellets were resuspended and sonicated in the buffers indicated in S7 Table.

After centrifugation, soluble fractions of KnRAV, KnRAV-DBD, CaARF, CaARF-DBD and CaARF mutants were loaded on Dextrin-Sepharose (GE Healthcare) column previously equilibrated in buffer A (S7 Table). After column washing, proteins were eluted in buffer A with maltose 10 mM (S6 Table).

PB1 domains of KnRAV and CaARF as well as full-length proteins ARF2, ARF5 and ARF10 were purified on Nickel-Sepharose (GE Healthcare) columns previously equilibrated in the appropriate buffers (S6 Table). After protein binding, columns were washed with 30 mM imidazole to remove all proteins non-specifically bound to the column. Proteins were eluted in the corresponding buffer containing 300 mM imidazole (S6 Table). His-tags of PB1 domains were cleaved by TEV protease (5% w/w) overnight at 4°C followed by incubation at 20°C for 2 h for SEC-MALLS experiments.

AtTPL202 and mutants were purified as explained in Martin-Arevalillo et al., 2017 [49]. Following purification step, all proteins were dialyzed for 15 h at 4°C in their purification buffers, frozen in liquid nitrogen and conserved at -80°C until used.

### EMSA DNA binding tests

DNA probes were artificially designed based on the DNA binding site for each TF (S8 Table) (Eurofins). Oligonucleotides for the sense strand were designed with an overhanging G in 5’ that allows the labelling of the DNA (S8 Table). Annealing of the oligonucleotides and Cy5-labelling of the probes were performed as described in Stigliani et al.,(2019) [19]. Electrophoretic Mobility Shift Assays (EMSA), were done on native 2% agarose gels prepared with TBE buffer 0.5X. Gels were pre-run in TBE buffer 0.5X at 90 V for 90 min at 4°C. Protein-DNA mixes contained Salmon and Herring Sperm competitor DNA (final concentration 0.07 mg/ml) and labelled DNA (final concentration 20 nM) in the interaction buffer (20 mM HEPES pH 7.8; 50 mM KCl; 100 mM Tris-HCl pH 8.0, 2.5% glycerol; 1 mM DTT). Mixes were incubated in darkness for 30 min at 4°C and next loaded in the gels. Gels were run for 1 hour at 90 V at 4°C in TBE 0.5X. DNA-protein interactions were visualized with Cy5-exposition filter (Biorad ChemiDoc MP Imaging System).

### Co-purification protein-protein interaction tests

For protein-protein interaction analyses, complexes between potential interaction partners were first formed by mixing MBP-tagged CaARF (wt or mutants) (90 μg) with His-tagged AtTPL202 (and mutants) (70 μg) in CAPS 20 mM pH 9.6; Tris-HCl 100 mM pH 8; NaCl 50 mM; TCEP 1 mM buffer for 1 h at 4°C. Complexes formed were fixed through the MBP tag to Dextrin-Sepharose columns previously equilibrated with CAPS 20 mM pH 9.6; Tris-HCl 100 mM pH 8; NaCl 50 mM; TCEP 0.1 mM buffer. After incubation of the complexes with Dextrin-Sepharose for 30 min at 4°C, nonspecific interactions were removed by a washing step with the same buffer. Protein complexes were eluted with 200 μl of the same buffer containing 10 mM of maltose. MBP was used as control for unspecific interactions. The eluted fractions were analysed by SDS-page polyacrylamide gel electrophoresis 12%.

### SEC-MALLS Native molecular mass determination

Molecular weights were determined by Size-Exclusion Chromatography-Multi Angle Light Scattering (SEC-MALLS) on an analytical Superdex-S200 increase (GE Healthcare) connected to an in-line MALLS spectrometer (DAWN HELEOS II, Wyatt Instruments). Analytical size exclusion chromatography was performed at 25°C at a rate of 0.5 mL/min for untagged PB1 domains resulting from TEV cleavage. Untagged KnRAV-PB1 MW determination was carried out in CAPS 100 mM pH 9.6; TCEP 1mM buffer, whereas Tris-HCl 20 mM pH 8; TCEP 1 mM was used for untagged CaARF-PB1 and AtARF5. The refractive index measured with in-line refractive index detector (Optirex, Wyatt Instruments) was used to follow the differential refractive index relative to the solvent. Molecular masses calculation was done with the Debye model using ASTRA version 5.3.4.20 (Wyatt Instruments) and a theoretical dn/dc value of 0.185 mL/g.

## Supporting information

S1 Fig Land plants evolutionary line from charophyte algae. Acquisition of more complex structures and features similar to those found in land plants is observed along charophytes evolutionary line (Adapted from [9]).
(PPTX)

S2 Fig Double AuxREs possible configurations. ARFs binding sites are double sites which can be Direct Repeats (DRs), with the binding sites located in the same DNA brand, Everted Repeats (ER) or Inverted Repeats (IR), with the binding sites in different DNA brands.
(PPTX)

S3 Fig ARF-DBDs alignment. Predicted charophyte ARF-DBDs aligned to DBDs of classes A (A.tha-5, 6, 7, 8, 19 and M.pol-1), B (A.tha-1, 2–4, 9, 11–15, 18, 20–22 and M.pol-2) and C (A.tha-10, 16 and 17 and M.pol-3) ARFs from *A*. *thaliana* and *M*. *polymorpha*. Class-C ARFs present an insertion in the DBD that is located inside the second part of the dimerization domain (DDII) (underlined) described for the ARFs (in between residues 260 and 280 in the alignment, referenced to A.tha-5). In agreement, ancestral ARFs sharing this insertion (C.atm, Entr, N.mir, C.irr, C.scu, M.end, S.pra) were classed with class-C ARFs, whereas proto-ARFs lacking this insertion (C. orb and Moug) belonged to class A/B (See phylogeny in Supplemental S4 Fig). Note that the M. vir sequence [1] does not contain the consensus B3 DNA binding sequence. Abbreviations used in the alignment: A.tha, *A*. *thaliana*; M.pol, *M*. *polymorpha*; M.vir, *M*. *viride*; C.atm, *C*.*atmophyticus*; Entr, *Entransia*; N.mir, *N*. *mirabilis*; C.irr, *C*. *irregularis*; C.scu, *C*. *scutata*; C.orb, *C*. *orbicularis*; Moug, *Mougeotia*; M.end, *M*. *endlicheranium*; S.pra, *S*. *pratensis*. Discontinuous underlines mark the regions involved in dimerization (DDI and DDII); violet underline marks B3 domain; black stars indicate the residues implicated in the interaction with AuxREs. Arrows point at C.atm and M.vir sequences. The incomplete sequence of the DBD of the class C ARF from *C*. *orbicularis* (GBSL01007362) was not added in the alignment.
(PPTX)

S4 Fig Residues conservation in KnRAV-DBD. **A**, B3^RAV^ domains alignment. Black stars indicate residues implicated in the interaction with DNA. WNSSQS, amino acids characteristic of B3^RAV^ TFs [45], are conserved in KnRAV (residues numbering referred to *A*.*thaliana* RAV1, A.tha-RAV1). **B**, AP2 domains alignment. Black stars indicate residues implicated in the interaction with DNA [53] (residues numbering referred to *A*. *thaliana* ERF1). Abbreviations used in the alignment: A.tha, *A*. *thaliana*; M.pol, *M*. *polymorpha*; K.nit, *K*. *nitens*.
(PPTX)

S5 Fig Phylogenetic classification of proto-ARFs in charophyte organisms. Maximum likelihood tree (built with MEGA software from DBD sequences) showing ARF evolutionary clades (A, B, C and A/B). Bootstrap values are shown next to branches. Abbreviations: A.tha, *A*. *thaliana*; M.pol, *M*. *polymorpha*; M.vir, *M*. *viride;* C.atm, *C*. *atmophyticus*; Entr, *Entransia*; N.mir, *N*. *mirabilis*; C.orb, *C*. *orbicularis*; C.irr, *C*. *irregularis*; C.scu, *C*. *scutata*; Moug, *Mougeotia*; M.end, *M*. *endlicheranium*; S.pra, *S*. *pratensis*. The incomplete sequence of the DBD of the class C ARF from *C*. *orbicularis* (GBSL01007362) was not added in the analysis.
(PPTX)

S6 Fig Different evolution hypotheses. **A.** ARF C and A/B originated from a common ancestor that had already diverged in an early charophyte and evolved independently in later clades, with subsequent losses in different clades/species. **B.** The presence of ARF C homologues from the first clades of charophytes evolutionary line suggests this subfamily or a closely-related one (C-like), as the common ancestor for current charophycean A/B and C ARFs. In both scenarios duplication of A/B into A and B happened in land plants. **C.** Phylogenetic tree generated by Maximum likehood (phylogeny.fr [54,55]) that supports charophytes C clade as ancestor of charophyte and land plants ARF subfamilies.
(PPTX)

S7 Fig Modelled DBDs from charophyte ancestral class C ARFs. Modelled structures superposed to ARF1 DBD structure (4LDX, in grey [46]). **A**, *Entransia* ARF-DBD, model in red. **B**, *N*. *mirabilis* ARF-C-DBD, model in green. **C**, *C*. *scutata* ARF-DBD, model in blue. **D**, *M*. *endlicheranium* ARF-DBD, model in yellow. **E**, *S*. *pratensis* ARF-DBD, model in purple. Yellow dots indicate the site of the insertion characteristic of ARF-C class members positioned either inside or at the end of the helix belonging to the DDII, depending on the model.
(PPTX)

S8 Fig Charophyte TPL N-ter aligment. TPL homologues found in charophytes aligned to *A*. *thaliana* (A.tha) and *M*. *polymorpha* (M.pol) TPL N-ter. Residues involved in the interaction with EAR motifs indicated with a triangle. Residues involved in TPL tetramerization indicated with a star. In the alignment: C.atm, *C*.*atmophyticus*; Entr, *Entransia*; N.mir, *N*. *mirabilis*; C.irr, *C*. *irregularis*; C.scu, *C*. *scutata*; C.orb, *C*. *orbicularis*; Moug, *Mougeotia*; M.end, *M*. *endlicheranium*; S.pra, *S*. *pratensis*.
(PPTX)

S1 Table Different B3 subfamilies and their DNA specificities. B3 domains were classed into B3^ABI3^ (not reported in this manuscript), B3^RAV^ and B3^ARF^ subfamilies according to the residues present in the predicted DNA-interacting loop. Indicated in the table the characteristic amino acidic sequence of the DNA-interacting loop and the DNA binding sequence for each B3 subfamily [45,56].
(DOCX)

S2 Table RAV and ARF sequences identified in the OneKP [11], Marchantia.info* [1,40], or *Klebsormidium nitens* genome** [7] databases. (DOCX)

S3 Table SEC-MALLS molecular weight determination of KnRAV-PB1, CaARF-PB1 and AtARF5-PB1 domains. (DOCX)

S4 Table TPL homologues found in charophyte organisms. Accession numbers for transcripts or proteins and the databases used for each search are indicated. Amino acidic sequences were obtained by transcripts translation, except for *K*.*nitens-*RAV protein, obtained from PlantTFDB. Predicted domains are indicated with a tick.
(DOCX)

S5 Table ARF Charophytes potential EAR motifs. Accession numbers for transcripts or proteins and the databases used for each search are indicated. Potential EAR motifs in the Middle Regions (MR) were searched for each protein, with the MR corresponding to the sequence in between the DBD domains and the PB1 domains. Possible EAR motifs were identified as potential TPL-recruitment sites based on the EAR/EAR-like motifs described in TPL interactome publication [35,36].
(DOCX)

S6 Table Primers for Domains amplification. Note that CaARF DBD and PB1 domains were isolated from CaARF through restriction sites introduced in CaARF synthetic cDNA (Thermofisher).
(DOCX)

S7 Table Buffers used for purification. (DOCX)

S8 Table DNA sequence probes used for EMSA assays. Sequences in bold indicating the binding sites and in italic the mutated sites.
(DOCX)
