# Supplementary figures and images for: Evolution of the Auxin Response Factors from charophyte ancestors
Source: PLoS Genet. 2019 Sep 25;15(9):e1008400. doi: 10.1371/journal.pgen.1008400 (PMC6797205; doi:10.1371/journal.pgen.1008400)

## Slide 1
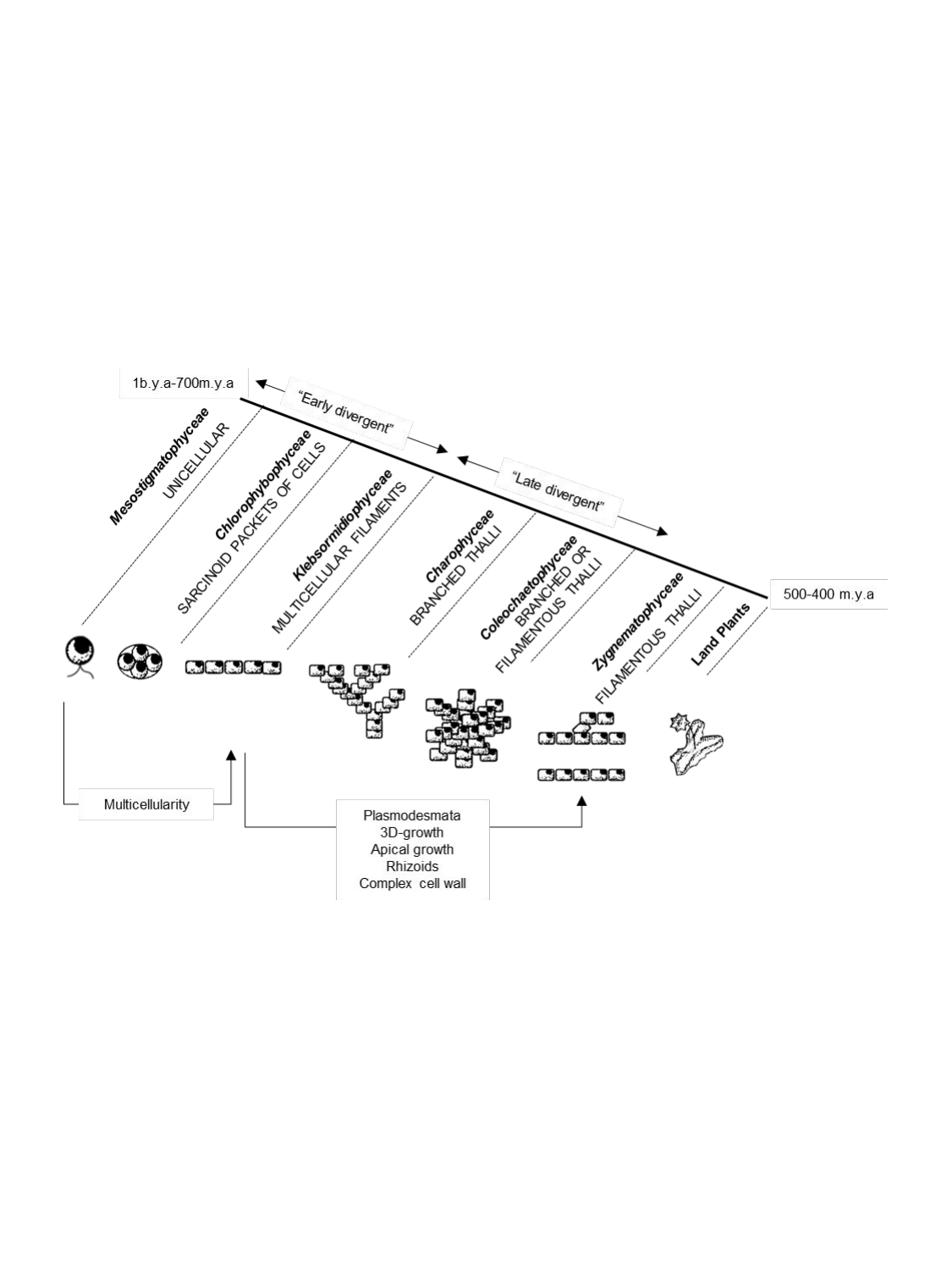

Supplement: S1 Fig — Acquisition of more complex structures and features similar to those found in land plants is observed along charophytes evolutionary line (Adapted from [9]). (PPTX) [file pgen.1008400.s001.pptx]

## Slide 1
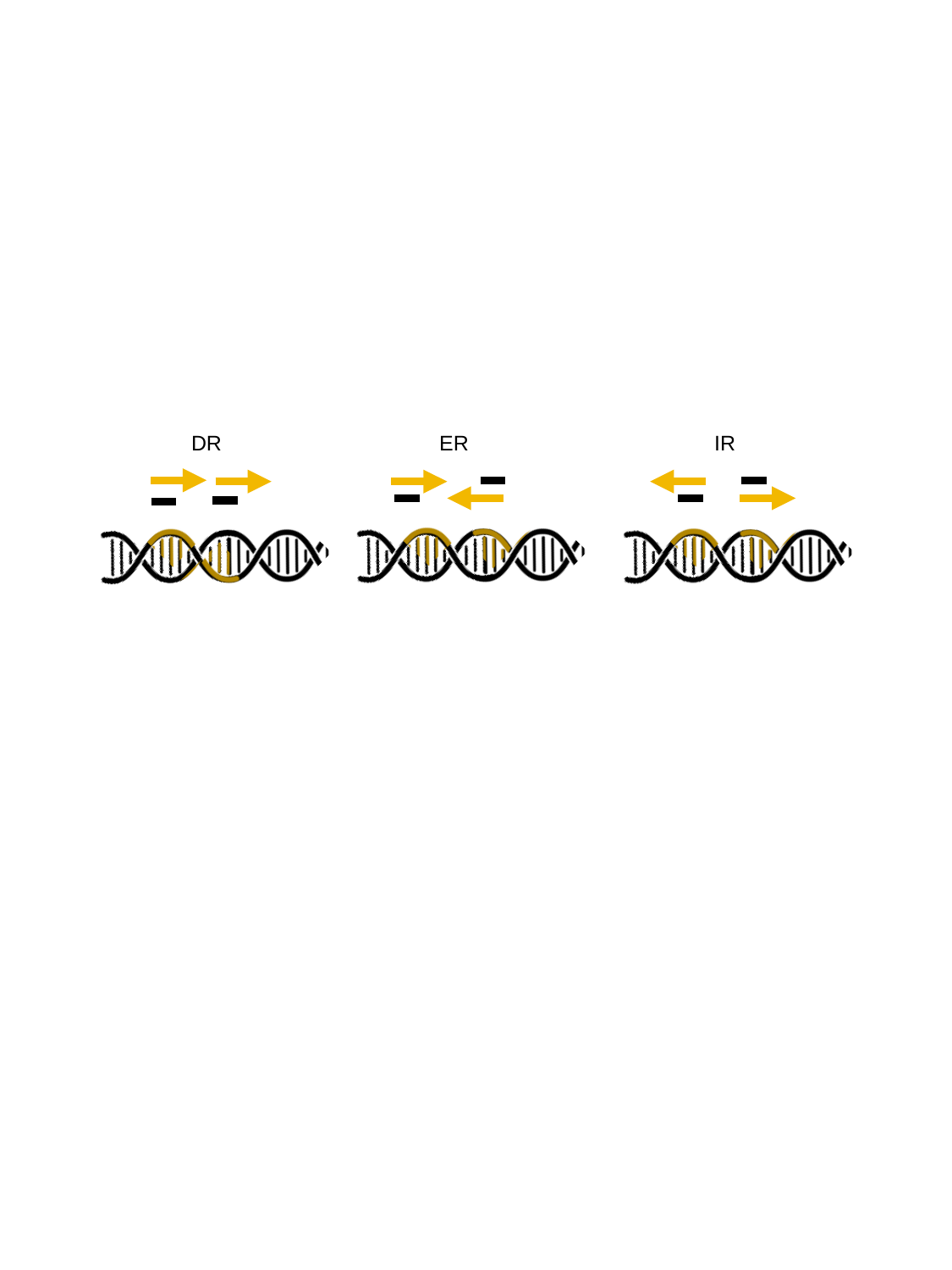

DR
ER
IR

Supplement: S2 Fig — ARFs binding sites are double sites which can be Direct Repeats (DRs), with the binding sites located in the same DNA brand, Everted Repeats (ER) or Inverted Repeats (IR), with the binding sites in different DNA brands. (PPTX) [file pgen.1008400.s002.pptx]

## Slide 1
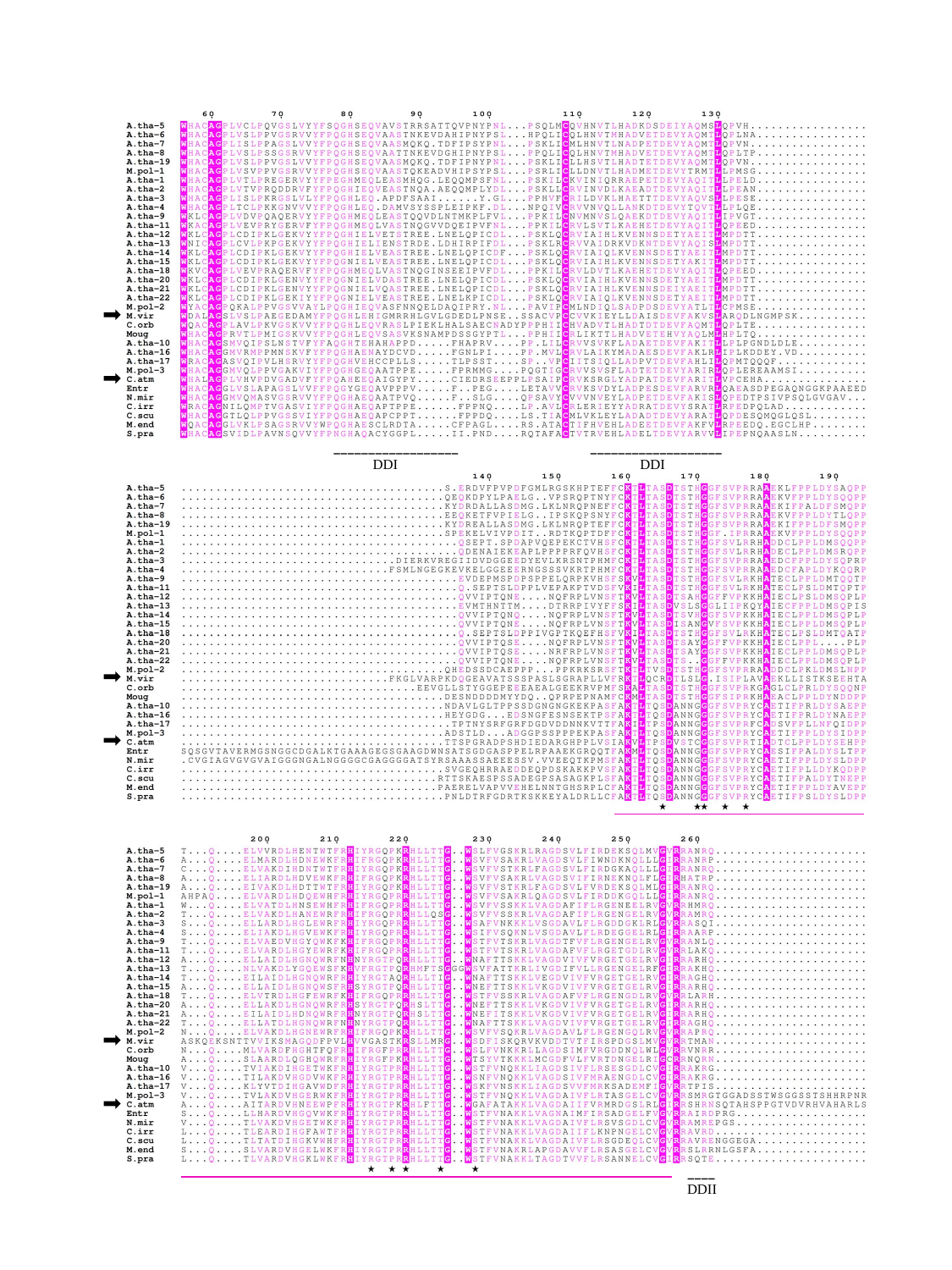

DDI
DDI
DDII

## Slide 2
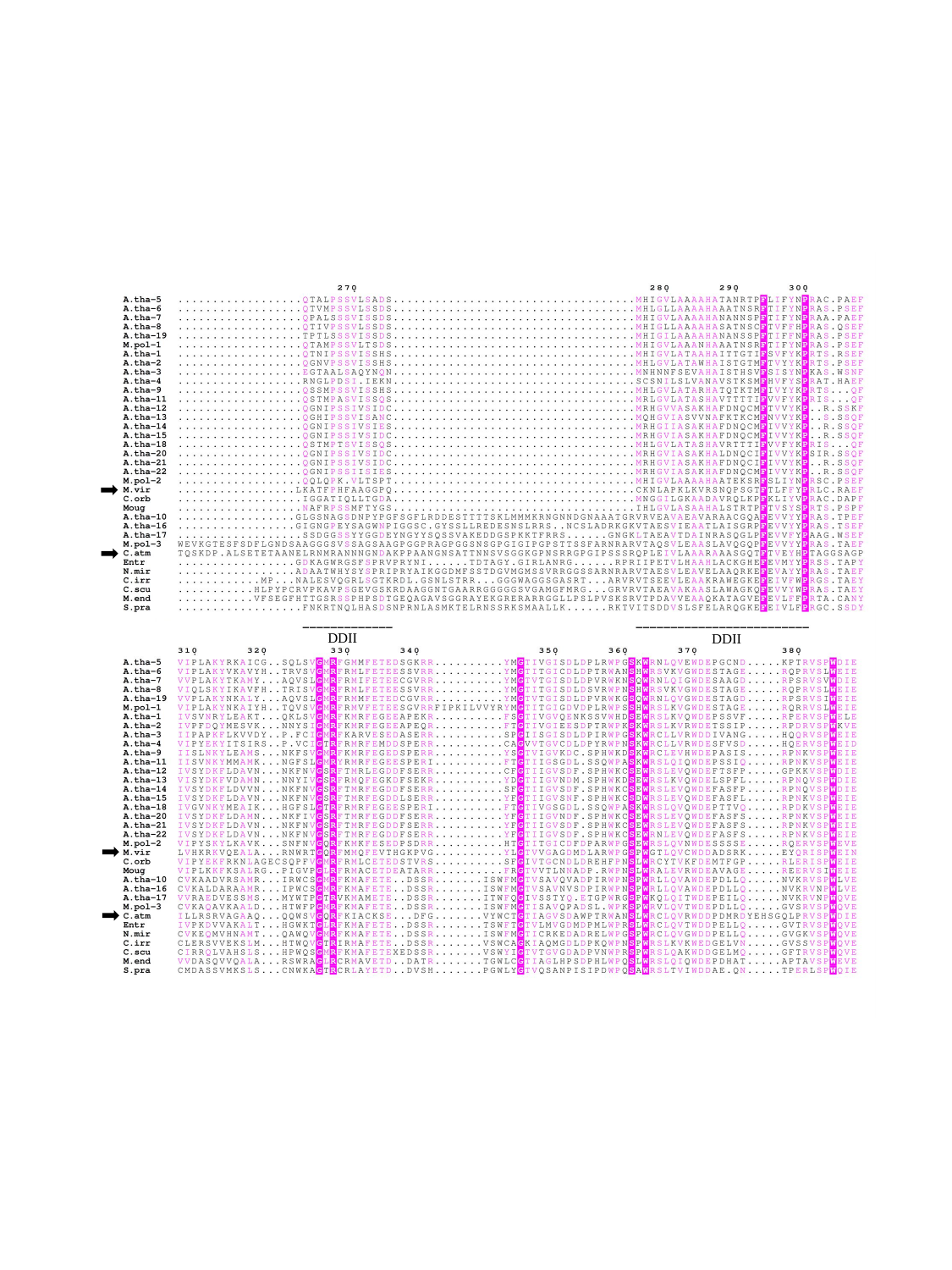

DDII
DDII

Supplement: S3 Fig — Predicted charophyte ARF-DBDs aligned to DBDs of classes A (A.tha-5, 6, 7, 8, 19 and M.pol-1), B (A.tha-1, 2–4, 9, 11–15, 18, 20–22 and M.pol-2) and C (A.tha-10, 16 and 17 and M.pol-3) ARFs from A. thaliana and M. polymorpha. Class-C ARFs present an insertion in the DBD that is located inside the second part of the dimerization domain (DDII) (underlined) described for the ARFs (in between residues 260 and 280 in the alignment, referenced to A.tha-5). In agreement, ancestral ARFs sharing this insertion (C.atm, Entr, N.mir, C.irr, C.scu, M.end, S.pra) were classed with class-C ARFs, whereas proto-ARFs lacking this insertion (C. orb and Moug) belonged to class A/B (See phylogeny in Supplemental S4 Fig). Note that the M. vir sequence [1] does not contain the consensus B3 DNA binding sequence. Abbreviations used in the alignment: A.tha, A. thaliana; M.pol, M. polymorpha; M.vir, M. viride; C.atm, C.atmophyticus; Entr, Entransia; N.mir, N. mirabilis; C.irr, C. irregularis; C.scu, C. scutata; C.orb, C. orbicularis; Moug, Mougeotia; M.end, M. endlicheranium; S.pra, S. pratensis. Discontinuous underlines mark the regions involved in dimerization (DDI and DDII); violet underline marks B3 domain; black stars indicate the residues implicated in the interaction with AuxREs. Arrows point at C.atm and M.vir sequences. The incomplete sequence of the DBD of the class C ARF from C. orbicularis (GBSL01007362) was not added in the alignment. (PPTX) [file pgen.1008400.s003.pptx]

## Slide 1
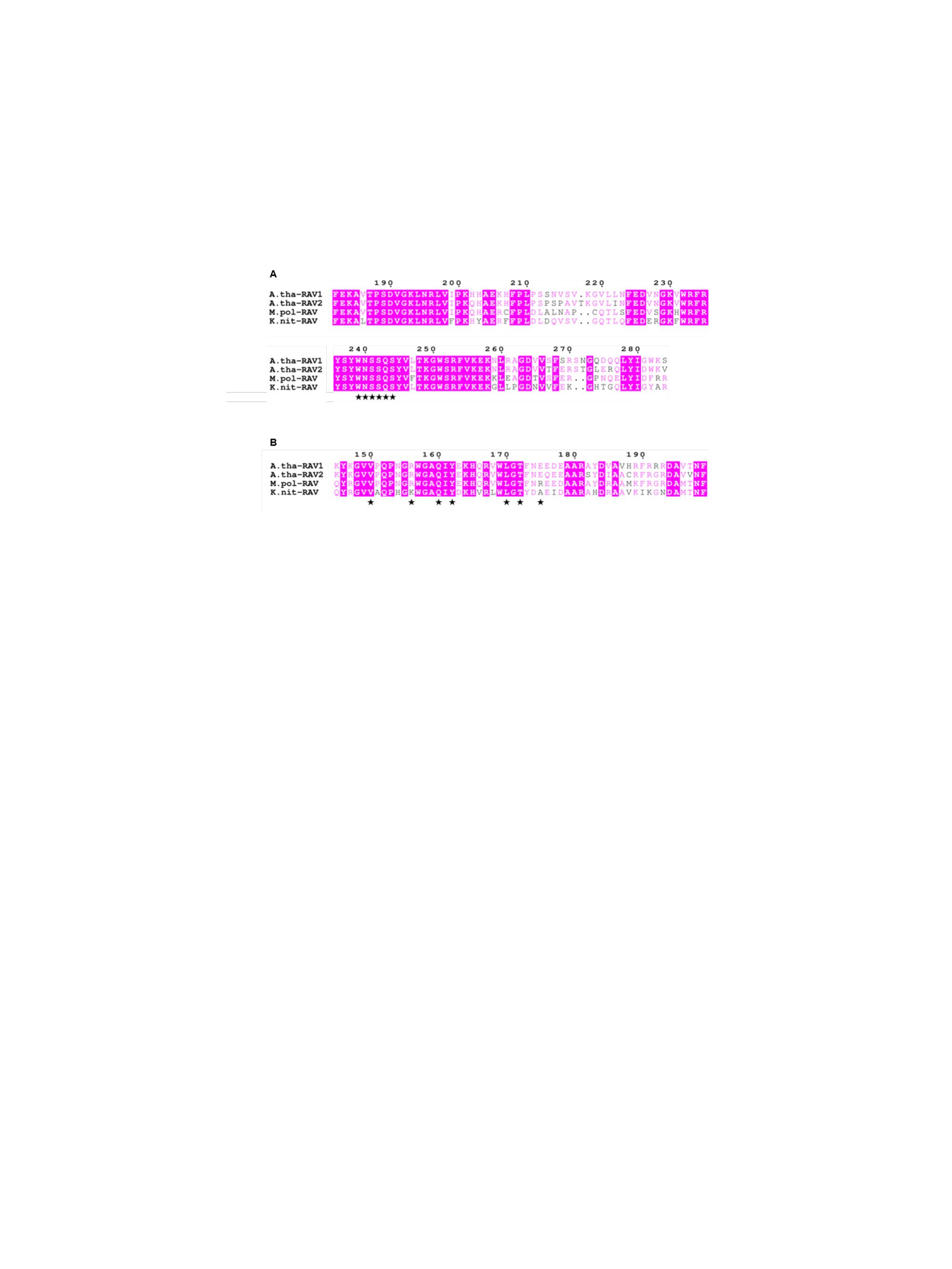

Supplement: S4 Fig — A, B3RAV domains alignment. Black stars indicate residues implicated in the interaction with DNA. WNSSQS, amino acids characteristic of B3RAV TFs [45], are conserved in KnRAV (residues numbering referred to A.thaliana RAV1, A.tha-RAV1). B, AP2 domains alignment. Black stars indicate residues implicated in the interaction with DNA [53] (residues numbering referred to A. thaliana ERF1). Abbreviations used in the alignment: A.tha, A. thaliana; M.pol, M. polymorpha; K.nit, K. nitens. (PPTX) [file pgen.1008400.s004.pptx]

## Slide 1
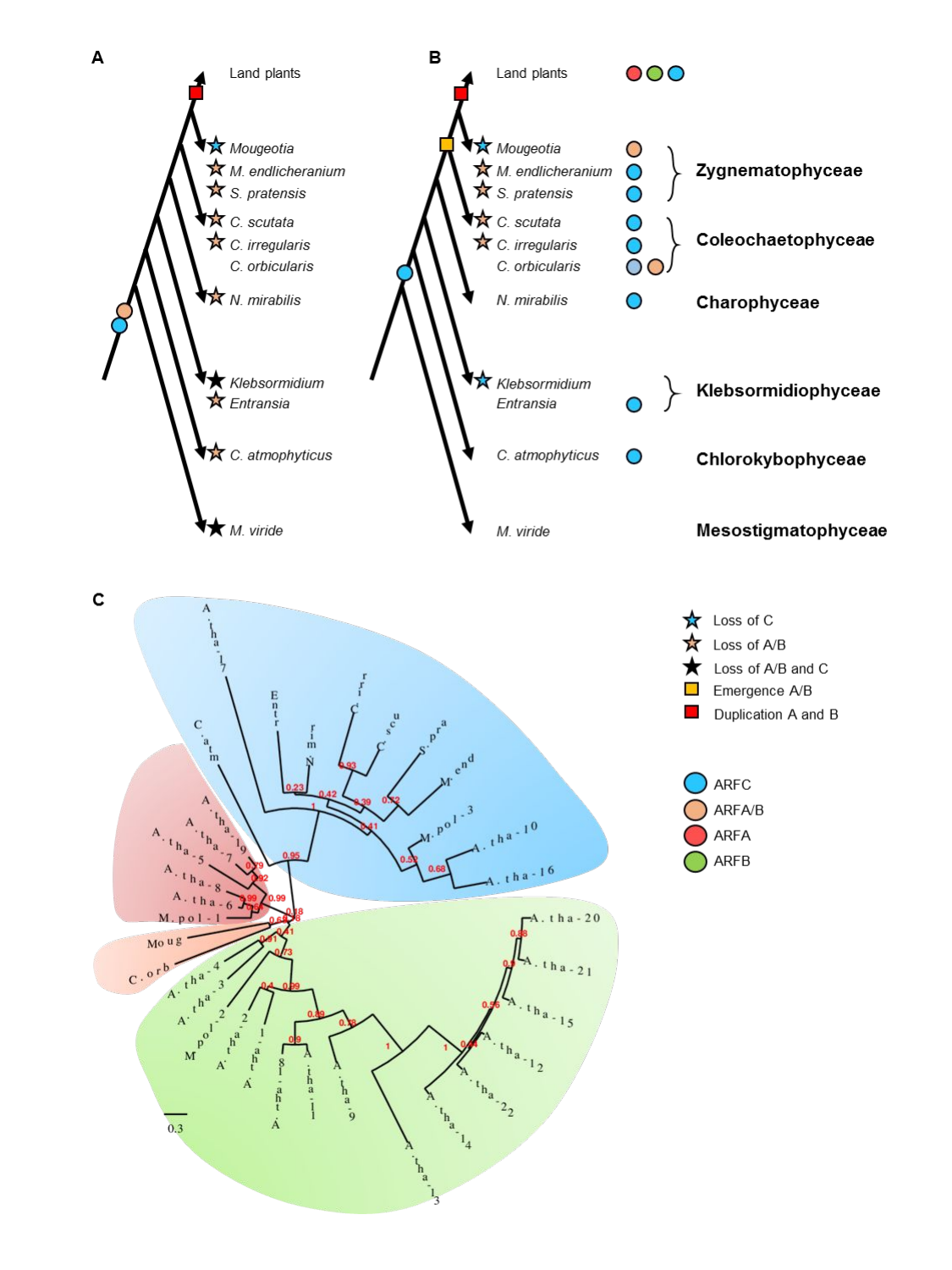

Supplement: S6 Fig — A. ARF C and A/B originated from a common ancestor that had already diverged in an early charophyte and evolved independently in later clades, with subsequent losses in different clades/species. B. The presence of ARF C homologues from the first clades of charophytes evolutionary line suggests this subfamily or a closely-related one (C-like), as the common ancestor for current charophycean A/B and C ARFs. In both scenarios duplication of A/B into A and B happened in land plants. C. Phylogenetic tree generated by Maximum likehood (phylogeny.fr [54,55]) that supports charophytes C clade as ancestor of charophyte and land plants ARF subfamilies. (PPTX) [file pgen.1008400.s006.pptx]

## Slide 1
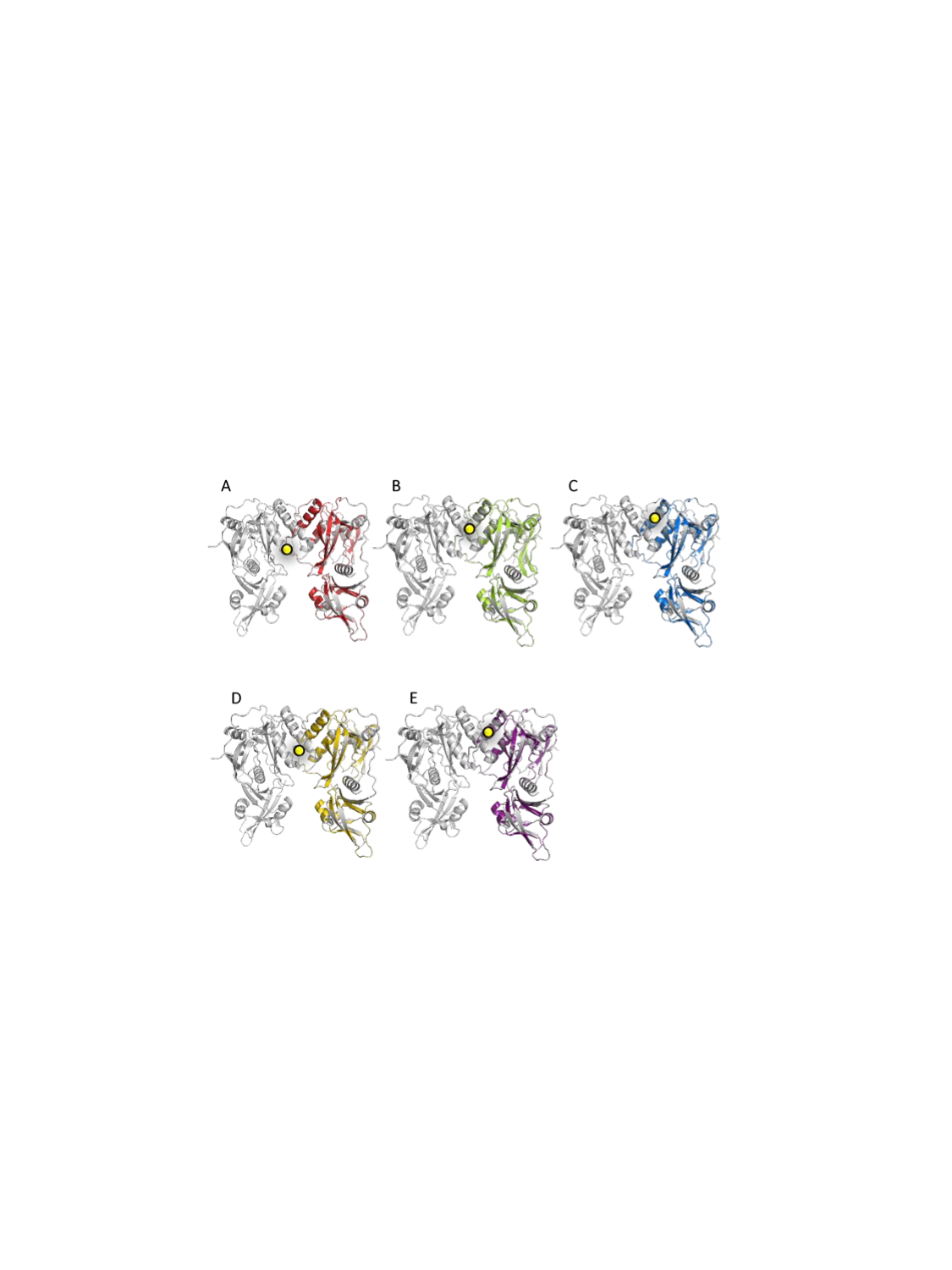

Supplement: S7 Fig — Modelled structures superposed to ARF1 DBD structure (4LDX, in grey [46]). A, Entransia ARF-DBD, model in red. B, N. mirabilis ARF-C-DBD, model in green. C, C. scutata ARF-DBD, model in blue. D, M. endlicheranium ARF-DBD, model in yellow. E, S. pratensis ARF-DBD, model in purple. Yellow dots indicate the site of the insertion characteristic of ARF-C class members positioned either inside or at the end of the helix belonging to the DDII, depending on the model. (PPTX) [file pgen.1008400.s007.pptx]

## Slide 1
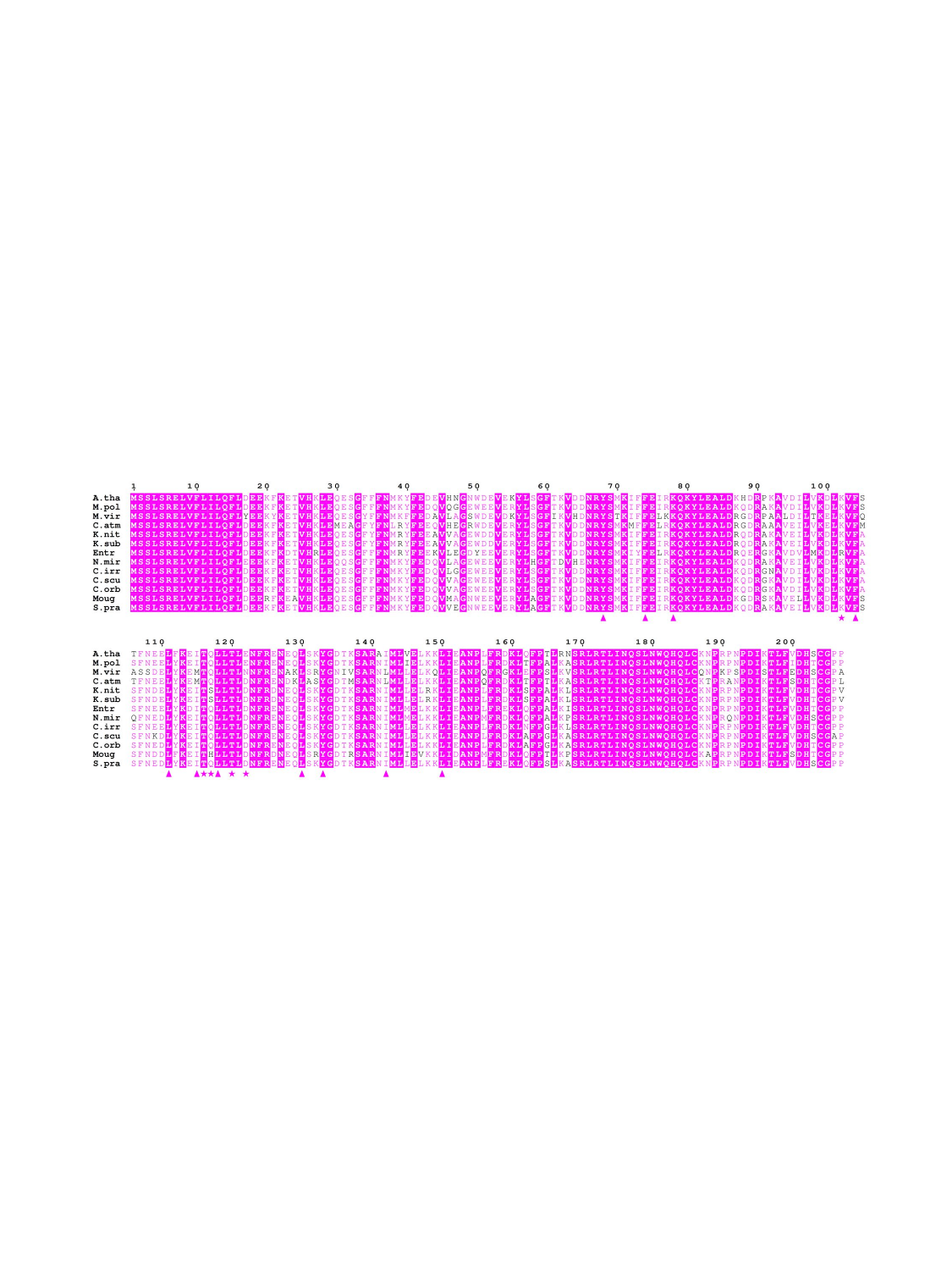

Supplement: S8 Fig — TPL homologues found in charophytes aligned to A. thaliana (A.tha) and M. polymorpha (M.pol) TPL N-ter. Residues involved in the interaction with EAR motifs indicated with a triangle. Residues involved in TPL tetramerization indicated with a star. In the alignment: C.atm, C.atmophyticus; Entr, Entransia; N.mir, N. mirabilis; C.irr, C. irregularis; C.scu, C. scutata; C.orb, C. orbicularis; Moug, Mougeotia; M.end, M. endlicheranium; S.pra, S. pratensis. (PPTX) [file pgen.1008400.s008.pptx]
