# Supplementary material for: Evolution of the Auxin Response Factors from charophyte ancestors
Source: PLoS Genet. 2019 Sep 25;15(9):e1008400. doi: 10.1371/journal.pgen.1008400 (PMC6797205; doi:10.1371/journal.pgen.1008400)
Supplement: S5 Fig — Maximum likelihood tree (built with MEGA software from DBD sequences) showing ARF evolutionary clades (A, B, C and A/B). Bootstrap values are shown next to branches. Abbreviations: A.tha, A. thaliana; M.pol, M. polymorpha; M.vir, M. viride; C.atm, C. atmophyticus; Entr, Entransia; N.mir, N. mirabilis; C.orb, C. orbicularis; C.irr, C. irregularis; C.scu, C. scutata; Moug, Mougeotia; M.end, M. endlicheranium; S.pra, S. pratensis. The incomplete sequence of the DBD of the class C ARF from C. orbicularis (GBSL01007362) was not added in the analysis. (PPTX) [file pgen.1008400.s005.pptx]

## Slide 1
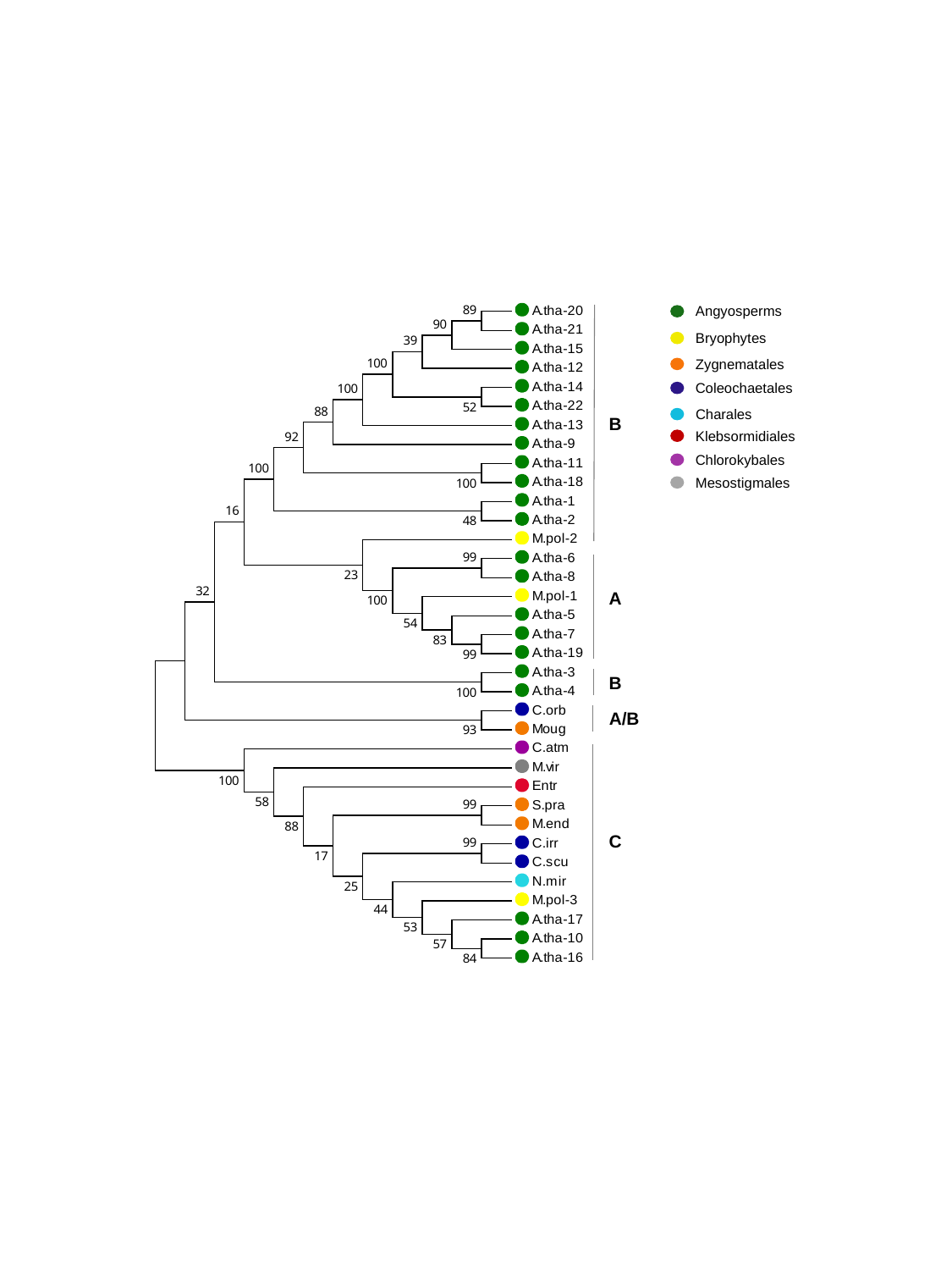

Angyosperms
Bryophytes
Zygnematales
Coleochaetales
Charales
B
Klebsormidiales
Chlorokybales
Mesostigmales
A
B
A/B
C
