# Supplementary material for: Evolution of the Auxin Response Factors from charophyte ancestors
Source: PLoS Genet. 2019 Sep 25;15(9):e1008400. doi: 10.1371/journal.pgen.1008400 (PMC6797205; doi:10.1371/journal.pgen.1008400)
Supplement: S1 Table — B3 domains were classed into B3ABI3 (not reported in this manuscript), B3RAV and B3ARF subfamilies according to the residues present in the predicted DNA-interacting loop. Indicated in the table the characteristic amino acidic sequence of the DNA-interacting loop and the DNA binding sequence for each B3 subfamily [45,56]. (DOCX) [file pgen.1008400.s009.docx]

| B3-type | Residues mediating specific DNA binding | DNA binding sequence |
| --- | --- | --- |
| ABI3 | WPNNKSR | 5’ CATGCA 3’ |
| RAV | WN/RSSQS | 5’ CACCTG 3’ |
| ARF | RGQ/TPK/RR | 5’ TGTCnn 3’ |
