# Supplementary material for: Evolution of the Auxin Response Factors from charophyte ancestors
Source: PLoS Genet. 2019 Sep 25;15(9):e1008400. doi: 10.1371/journal.pgen.1008400 (PMC6797205; doi:10.1371/journal.pgen.1008400)
Supplement: S2 Table — (DOCX) [file pgen.1008400.s010.docx]

| **Chlorophytaceae** | **RAV** | **ARF** |
| --- | --- | --- |
| **Mesostigmatophyceae  *M. viride*** | GBSK01010633.1*  GBSK01014943.1*  KYIO-2008649 | GBSK01006108.1* |
| **Chlorokybophyceae *C. atmophyticus*** | AZZW-2021745 | AZZW-2021616 |
| **Klebsormidiophyceae *K. nitens***    ***Entransia*** | kfl00094_0070** |  |
|  | BFIK-2030872 | BFIK-2028190* |
| **Charophyceae *N. mirabilis*** | GBST01061309.1* | GBST01078830.1* |
| **Coleochaetophyceae *C. scutata***    ***C. irregularis***    ***C. orbicularis*** | VQBJ-2009355* | VQBJ-2004071 |
|  |  | QPDY-2028497 |
|  |  | GBSL01031616.1*  GBSL01007362 * |
| **Zygnematophyceae *Mougeotia***    ***M. endlicheranium***    ***S. pratensis*** | ZRMT-2007919 | ZRMT-2006773 |
|  | WDCW-2005906 | WDCW-2048330 |
|  | GBSM01008321.1* | GBSM01024076.1* |
