# Supplementary material for: Evolution of the Auxin Response Factors from charophyte ancestors
Source: PLoS Genet. 2019 Sep 25;15(9):e1008400. doi: 10.1371/journal.pgen.1008400 (PMC6797205; doi:10.1371/journal.pgen.1008400)
Supplement: S3 Table — (DOCX) [file pgen.1008400.s011.docx]

| **KnRAV-PB1** | **Main peak**  **MW (KDa)//**  **Oligomeric state** | **Small peak**  **MW (KDa)//**  **Oligomeric state** | **CaARF-PB1** | **MW (KDa)//**  **Oligomeric state** | **AtARF5-PB1** | **MW (KDa)//**  **Oligomeric state** |
| --- | --- | --- | --- | --- | --- | --- |
| 0.625 mg/ml; 69μM | 14.2±10.8%  1.57 monomoers |  | 0.625 mg/ml;  52μM | 18.8±5.8%  1.56 monomers |  |  |
| 1.25 mg/ml;  138μM | 17.8±2.6%  1.97 monomers | 35.1±5.9%  3.9 monomers | 1.25 mg/ml;  104μM | 21.6±5.3%  1.8 monomers |  |  |
| 2.5 mg/ml;  277μM | 19.7±1.2%  2.19 monomers | 33.1±2.4%  3.68 monomers | 2.5 mg/ml;  208μM | 26.9±1%  2.24 monomers | 3.1 mg/ml;  220μM | 82.5±7.2%  5.9 monomers |
| 5 mg/ml;  555μM | 28±10.8%  3.11 monomers | 49±1%  5.44 monomers | 5 mg/ml;  416μM | 35.8±0.4%  2.98 monomers | 5 mg/ml; 354μM | 99.2±5%  7 monomers |
