# Supplementary material for: Evolution of the Auxin Response Factors from charophyte ancestors
Source: PLoS Genet. 2019 Sep 25;15(9):e1008400. doi: 10.1371/journal.pgen.1008400 (PMC6797205; doi:10.1371/journal.pgen.1008400)
Supplement: S4 Table — Accession numbers for transcripts or proteins and the databases used for each search are indicated. Amino acidic sequences were obtained by transcripts translation, except for K.nitens-RAV protein, obtained from PlantTFDB. Predicted domains are indicated with a tick. (DOCX) [file pgen.1008400.s012.docx]

|  | **Accession number** | **Database** | **LiSH/CTLH/CRA** | **First**  **WD40** | **Second**  **WD40** |
| --- | --- | --- | --- | --- | --- |
| *Mesostigma viride* | GBSK01064957.1 | Marchantia.info | ✔ |  |  |
| *Chlorokybus atmophyticus* | AZZW-2021890 | OneKP | ✔ | ✔ | ✔ |
| *Klebsormidium nitens* | kfl00881_0020_v1.1 | PlantTFDB | ✔ | ✔ | ✔ |
| *Klebsormidium subtile* | FQLP-2010078 | OneKP | ✔ | ✔ | ✔ |
| *Entransia* | BFIK-2004708 | OneKP | ✔ | ✔ | ✔ |
| *Nitella mirabilis* | GBST01062325.1 | Marchantia.info | ✔ | ✔ |  |
| *Coleochaete irregularis* | QPDY-2006907 | OneKP | ✔ | ✔ |  |
| *Coleochaete scutata* | VQBJ-2012007 | OneKP | ✔ | ✔ | ✔ |
| *Coleochaete orbicularis* | GBSL01014775.1 | Marchantia.info | ✔ | ✔ |  |
| *Mougeotia* | ZRMT-2005604 | OneKP | ✔ | ✔ |  |
| *Spirogyra pratensis* | GBSM01001798.1 | Marchantia.info | ✔ | ✔ | ✔ |
