# Supplementary material for: Evolution of the Auxin Response Factors from charophyte ancestors
Source: PLoS Genet. 2019 Sep 25;15(9):e1008400. doi: 10.1371/journal.pgen.1008400 (PMC6797205; doi:10.1371/journal.pgen.1008400)
Supplement: S5 Table — Accession numbers for transcripts or proteins and the databases used for each search are indicated. Potential EAR motifs in the Middle Regions (MR) were searched for each protein, with the MR corresponding to the sequence in between the DBD domains and the PB1 domains. Possible EAR motifs were identified as potential TPL-recruitment sites based on the EAR/EAR-like motifs described in TPL interactome publication [35,36]. (DOCX) [file pgen.1008400.s013.docx]

|  | **Accession number** | **Database** | **Potential EAR motif in MR** |
| --- | --- | --- | --- |
| *Chlorokybus atmophyticus* | **AZZW-2021616** | OneKP | LPLLP-Partial LxLxL (LxL)  KLFGV |
| *Entransia* | BFIK-2028190 | OneKP | LALPL  MLFGV |
| *Nitella mirabilis* | GBST01078830.1 | Marchantia.info | LVPLL, Unknown |
| *Coleochaete irregularis* | QPDY-2028497 | OneKP |  |
| *Coleochaete scutata* | VQBJ-2004071 | OneKp | Rich-L region, multiple LLL repeats |
| *Coleochaete orbicularis* | GBSL01031616.1 | Marchantia.info |  |
| *Mougeotia* | ZRMT-2006773 | OneKP | LDLALRL  Multiple partial LxLxL (LxL) |
| *Mesotaenium endlicheranium* | WDCW-2048330 | OneKP | LDLR-Partial LxLxL (LxL)  LLQQL-Partial LLLxL |
| *Spirogyra pratensis* | GBSM01024076.1 | Marchantia.info | LSPLPLP- Partial LxLxL (LxL)  LMLAL  MLFGRV |
