# Supplementary material for: Evolution of the Auxin Response Factors from charophyte ancestors
Source: PLoS Genet. 2019 Sep 25;15(9):e1008400. doi: 10.1371/journal.pgen.1008400 (PMC6797205; doi:10.1371/journal.pgen.1008400)
Supplement: S6 Table — Note that CaARF DBD and PB1 domains were isolated from CaARF through restriction sites introduced in CaARF synthetic cDNA (Thermofisher). (DOCX) [file pgen.1008400.s014.docx]

| **Domain** | **DNA sequence (5’->3’)** |
| --- | --- |
| KnRAV-PB1 | TATACCATGGCAGAACAGCGTCCGCAGGTTAAATGT |
|  | GTGCTCGAGTTATTTGCGGATGAAGATACGGCT |
| KnRAV-DBD | TAATCTCGAGTTAACGTGCATAACCAATATACAGCTG |
|  | TATACCATGGCAGAACGCGGTCTGGAACAGGGCCTG |
