# Supplementary material for: Evolution of the Auxin Response Factors from charophyte ancestors
Source: PLoS Genet. 2019 Sep 25;15(9):e1008400. doi: 10.1371/journal.pgen.1008400 (PMC6797205; doi:10.1371/journal.pgen.1008400)
Supplement: S7 Table — (DOCX) [file pgen.1008400.s015.docx]

| **Protein** | **Sonication and Purification buffer** | **Elution Buffer** |
| --- | --- | --- |
| MBP-KnRAV-DBD,  MBP-CaARF,  MBP-CaARF-DBD | Buffer A (Tris-HCl 20mM pH8; NaCl 500mM; TCEP 1mM) | Buffer A containing 10 mM maltose |
| His-KnRAV-PB1 | Buffer B (CAPS 100mM pH 9.6; TCEP 1mM) | Buffer B containing 300 mM imidazol |
| His-CaARF-PB1 | Buffer C (Tris 20mM pH8; TCEP 1mM) | Buffer C containing 300 mM imidazol |
| His-AtARF5-PB1 | Buffer C | Buffer C containing 300 mM imidazol |
| MBPHis-ARF2,  MBPHis-ARF5,  MBPHis-ARF10 | Buffer A | Buffer A containing 300 mM imidazol |
