# Supplementary material for: Evolution of the Auxin Response Factors from charophyte ancestors
Source: PLoS Genet. 2019 Sep 25;15(9):e1008400. doi: 10.1371/journal.pgen.1008400 (PMC6797205; doi:10.1371/journal.pgen.1008400)
Supplement: S8 Table — Sequences in bold indicating the binding sites and in italic the mutated sites. (DOCX) [file pgen.1008400.s016.docx]

| **Probe** | **DNA sequence (5’->3’)** |
| --- | --- |
| ABI3 | GATACACGCT**CATGCA**CCAAAGGCGG |
| ARF | GATACACGCT**TGTCTC**CCAAAGGCGG |
| RAV | GATACACGCT**CACCTG**CCAAAGGCGG |
| AP2 | GATACACGCT**Caaca**CCAAAGGCGGA |
| AP2/RAV  AP2(-)/RAV  AP2/RAV(-)  AP2(-)/RAV(-) | Gcgg**caaca**ataCA**CACCTG**ACTCGA  Gcgg**ca***TGT*ataCA**CACCTG**ACTCGA  Gcggc**aaca**ataAACA*ata*GACTCGA  gcggc**a***tgt*ATACACA*ata*GACTCGA |
| DR5 | GATACACGCAA**TGTCGG**CCTTT**TGTCGG**TTCCACTCA |
| ER4 | GATACACGCT**TGTCGG**CAAG**CCGACA**ACCACTCA |
| ER5 | GATACACGCT**TGTCGG**CAAAG**CCGACA**ACCACTCA |
| ER6 | GATACACGCT**TGTCGG**CCAAGG**CCGACA**ACCACTCA |
| ER7 | GATACACGCT**TGTCGG**CCAAAGG**CCGACA**ACCACTCA |
| ER8 | GATACACGCT**TGTCGG**CCAAAAGG**CCGACA**ACCACTCA |
| ER9 | GATACACGCT**TGTCGG**CCAAGAAGG**CCGACA**ACCACTCA |
| SS | GATACACGCT**TGTCGG**CCAAAGGC*GGTGT*ACCACTCA |
